# Prediction model of cervical lymph node metastasis based on clinicopathological characteristics of papillary thyroid carcinoma: a dual-center retrospective study

**DOI:** 10.3389/fendo.2023.1233929

**Published:** 2023-09-11

**Authors:** Wenji Liu, Die Zhang, Hui Jiang, Jie Peng, Fei Xu, Hongxin Shu, Zijian Su, Tao Yi, Yunxia Lv

**Affiliations:** ^1^ Department of Thyroid Surgery, Second Affiliated Hospital of Nanchang University, Nanchang, Jiangxi, China; ^2^ Second Clinical Medical College, Nanchang University, Nanchang, Jiangxi, China; ^3^ Medical Department, The First Clinical Medicine College, Nanchang University, Nanchang, Jiangxi, China; ^4^ Department of Otolaryngology, Yichun People’s Hospital, Yichun, Jiangxi, China

**Keywords:** papillary thyroid carcinoma, lymph node metastasis, preoperative serum thyroid stimulating hormone, clinicopathological characteristics, prediction model

## Abstract

**Background:**

The overall prevalence of papillary thyroid carcinoma (PTC) patients is expanding along with an ongoing increase in thyroid cancer incidence. Patients with PTC who have lymph node metastases have a poor prognosis and a high death rate. There is an urgent need for indicators that can predict lymph node metastasis (LNM) before surgery as current imaging techniques, such as ultrasonography, do not have sufficient sensitivity to detect LNM. To predict independent risk factors for Central lymph node metastasis (CLNM) or Lateral lymph node metastasis (LLNM), we therefore developed two nomograms based on CLNM and LLNM, separately.

**Methods:**

In two centers, the Second Affiliated Hospital of Nanchang University and Yichun People’s Hospital, we retrospectively analyzed clinicopathological characteristics of PTC patients. We utilized multivariate analysis to screen for variables that might be suspiciously related to CLNM or LLNM. Furthermore, we developed nomograms to graphically depict the independent risk valuables connected to lymph node metastasis in PTC patients.

**Result:**

Ultimately, 6068 PTC patients in all were included in the research. Six factors, including age<45, male, mETE, TSH>1.418, tumor size>4cm, and location (multicentric and lobe), were observed to be related to CLNM. Age<45, male, mETE (minimal extrathyroidal extension), multifocality, TSH≥2.910, CLNM positive, and tumor size>4cm were regarded as related risk factors for LLNM. The two nomograms developed subsequently proved to have good predictive power with 0.706 and 0.818 and demonstrated good clinical guidance functionality with clinical decision curves and impact curves.

**Conclusion:**

Based on the successful establishment of this dual-institution-based visual nomogram model, we found that some clinical features are highly correlated with cervical lymph node metastasis, including CLNM and LLNM, which will better help clinicians make individualized clinical decisions for more effectively rationalizing managing PTC patients.

## Introduction

Thyroid cancer, currently the fifth most prevalent cancer among American women, has recently seen a dramatic increase in rates, causing it to become the ninth most common cancer worldwide by 2020 (1, 2). The most prevalent category of thyroid cancer, known as papillary thyroid carcinoma (PTC), deriving from thyroid follicular epithelial cells, is well-differentiated and has a favorable prognosis compared with other subtypes such as medullary thyroid cancer and anaplastic thyroid cancer (3). PTC patients, a large population base with a good prognosis, generally have a 10-year survival rate of> 90% (4, 5).

Central lymph node metastasis (CLNM) and lateral lymph node metastasis (LLNM) refer to the metastasis of tumor cells to the cervical lymph nodes (level VI including pre-tracheal, paratracheal, pre-cricoid, and perithyroid lymph nodes) and cervical lymph nodes (level II-V) respectively (6). In PTC patients, LLNM usually occurs after CLNM in the development of cervical LNM (7). Compared with LLNM, CLNM is more common and occurs in the early stages of PTC, ranging from 11.7% to 63.8% of PTC patients (8). Relevant studies have shown that even in patients who have had a clinical examination and have no cervical lymph node metastases, the incidence of CLNM can range from 15.9% to 53% (9). LLNM occurs infrequently, ranging from 3.1% to 65.4% (median 19.6%), and usually occurs in the advanced stages of PTC (10). Besides, lymph node metastasis (LNM) is a vital prognostic indicator of PTC. Both CLNM and LLNM increase the risk of regional recurrence, while LLNM increases the risk of distant metastasis which cause a higher mortality (11). Therefore, lymph node dissection (LND) is very important in the surgical process of PTC.

It’s accepted that PTC patients with no distant metastasis can generally be cured by surgery or radiofrequency ablation, with 10-year cause-specific survival (96.8% for total thyroidectomy vs. 98.6% for lobectomy) (11). Thyroidectomy and cervical lymph node dissection are commonly recommended by clinicians for PTC patients with positive central or lateral lymph node metastasis on ultrasound and prophylactic lateral lymph node dissection (PLLND) is not recommended for patients with clinical LLNM negative. However, some studies have suggested that the number of positive CLNM may influence the emergence of occult LLNM (10, 12). What’s more, whether to perform prophylactic central lymph neck dissection (PCLND) in clinical CLNM-negative patients is still a matter of expert debate. While there are medical associations, such as the Chinese Thyroid Society and the Japanese Society of Endocrine Surgery, that support performing PCLND for patients with T1/T2 PTC patients with negative clinical lymph node metastasis (13), PCLND is considered to have a poor prognostic benefit and affects patient disease-free survival with the existence of some potential complications, such as permanent hypoparathyroidism, recurrent laryngeal nerve injury, etc (13, 14). However, the American Thyroid Association (ATA) guidelines suggest PCLND for patients with cancer recurrence, advanced primary tumors (T3 or T4), or clinically involved lateral neck nodes (cN1b) (15). Therefore, many surgeons remain skeptical toward prophylactic lymph neck dissection, compared to therapeutic lymph node dissection.

For cervical LNM, there are a few invasive diagnostic techniques, such as fine-needle aspiration biopsy (FNA) and fine-needle aspiration washout thyroglobulin (FNA-Tg) with higher evaluation reliability (16). However, lymph node metastatic thyroid cancer cannot be detected by non-invasive high-resolution ultrasonography (US) at a sufficiently sensitive rate, despite its significance in identifying aberrant lymph node metastasis (15). A paucity of evidence exists to affirm the effect of imaging techniques for preoperative diagnosis of LNM in PTC (17–19), and studies aimed to forecast the likelihood of LNM in PTC patients do not have consistent results or do not discuss the possibility of CLNM or LLNM, separately (12, 20, 21). It is necessary to further explore more effective methods for predicting the incidence of CLNM and LLNM in PTC patients.

In this retrospective research, total clinicopathological examination data of 6068 patients with PTC in the training and internal validation cohort and 582 patients with PTC in the external testing cohort were gathered in an attempt to build a model to predict the development of cervical LNM in PTC patients, including CLNM and LLNM, and to confirm the predictive ability of this model, which can aid clinical staff and patients in choosing treatment options and assessing the prognosis.

## Materials and methods

### Patient selection

In this dual-center study, we retrospectively investigated and collected data from PTC patients surgically treated in two hospitals from February 2011 to April 2022. The selected patients satisfy the following criteria: pathology-confirmed PTC, complete data baseline, clinicopathological characteristics (including the extrathyroidal extension of cancer, multifocality, tumor location, tumor size, and tumor biopsy data (including CLNM, LLNM)), preoperative laboratory data. Tumor size refers to the maximum diameter of a single tumor detected by using intraoperative pathology report, and in the case of multiple tumors, it is the maximum diameter of the largest tumor. Following the patient’s admission to the hospital, all preoperative laboratory data were collected the following morning (between 6:00 and 8:00 AM). Prior to sample collection, patients had to fast for eight hours. The serum was isolated right away. All data were meticulously documented.

**Figure 1:**
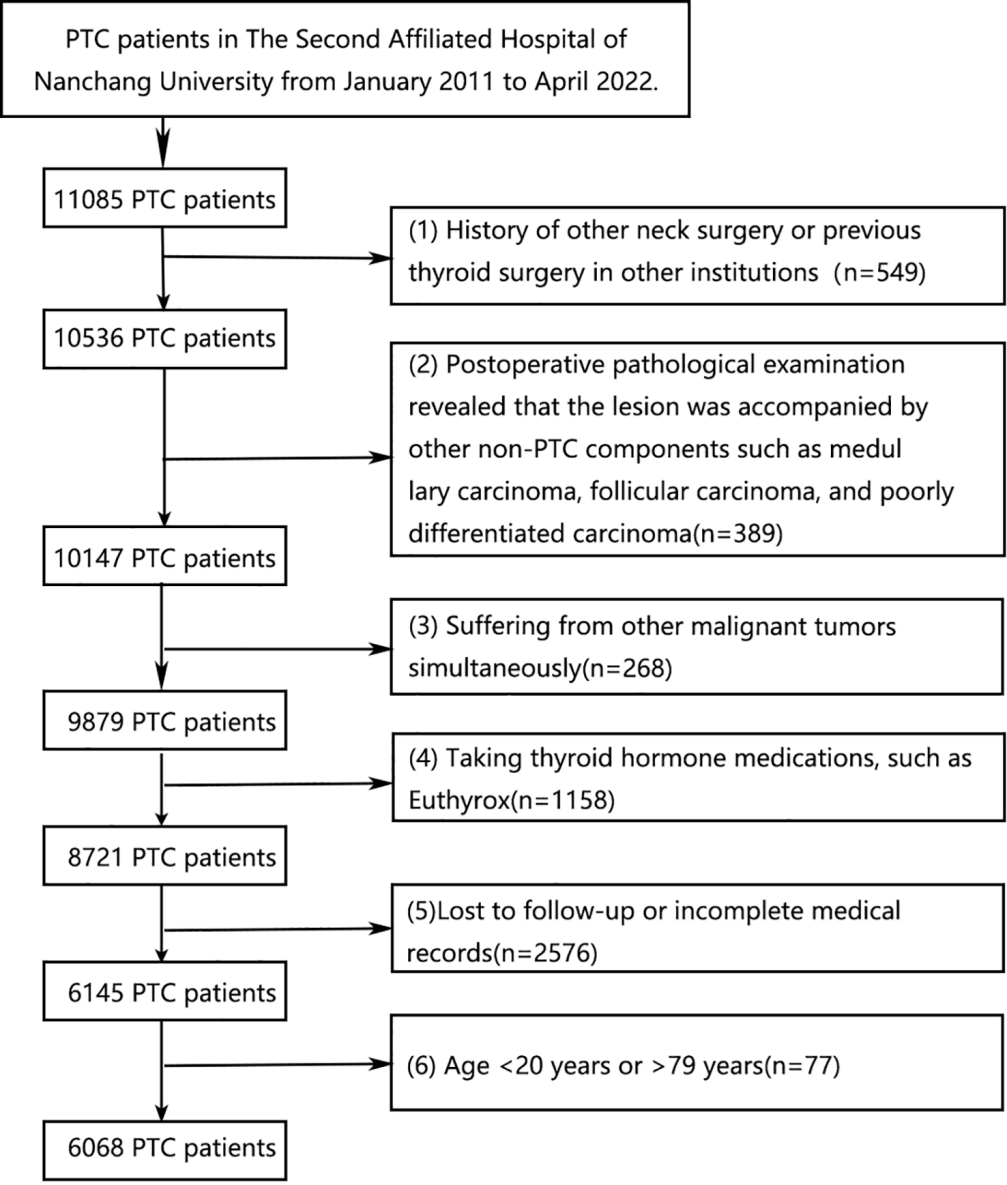
PTC patients exclusion flowchart.

The excluding criteria are as follows: (1) History of other neck surgery or previous thyroid surgery in other institutions; (2) Postoperative pathological examination revealed that the lesion was accompanied by other non-PTC components including medullary carcinoma, poorly differentiated carcinoma, and follicular carcinoma; (3) Suffering from other malignant tumors simultaneously; (4) Using thyroid hormone medications, such as Levothyroxine sodium; (5) Lost to follow-up or incomplete medical records; (6) Age<20 or >79 years old. 6649 PTC patients in total, including 6068 cases in the Second Affiliated Hospital of Nanchang University and 582 cases in the Yichun People’s Hospital, were enrolled in our study (**Figure 1**).

### Treatment

Before surgery, all of the study patients received the US to assess the health of their lymph nodes. According to statistics, a total of 4279 PTC patients admitted to the Second Affiliated Hospital of Nanchang University were negative for CLNM, 347 underwent central neck dissection (CLND) with CLNM negative, while 5345 were negative for LLNM, and 142 underwent lateral neck dissection LLND with LLNM negative. No recurrence and no lymphatic metastasis within one year of follow-up is considered lymph node negative. According to Chinese surgical guidelines for thyroid diagnosis and treatment, the standard procedure for patients with PTC in this study was thyroidectomy (14). Patients with PTC who were treated were routinely dissected ipsilateral central lymph nodes. Bilateral central lymph node dissection and a complete thyroidectomy were performed on patients who had bilateral PTC. Part of the patients with unilateral PTC with highly invasive or extrathyroid infiltration underwent total thyroidectomy and isthmus resection. lateral lymph node dissection (LLND) was performed if preoperative ultrasound or fine-needle biopsy confirmed lateral lymph node metastasis.

### Statistical analysis

6068 PTC patients were divided into the following two cohorts in a 7:3 ratio randomly: the training cohort (n = 4247) and the internal validation cohort (n = 1821). To clarify the baseline data and general characteristics of PTC patients, we used descriptive statistics. Numbers and percentages were used to represent categorical variables. Pearson’s chi-square test and Wilcoxon test were employed to verify the consistency of two cohorts after random grouping. In addition, the continuous variables failing the Kolmogorov-Smirnov test were considered not to satisfy the normal distribution and reported as medians (quartile 1, quartile 3).

In this study, risk factors for CLNM or LLNM have been evaluated and documented, and preoperative laboratory data (blood chemistry analysis) of serum TSH, FT3, FT4, and the ratio of FT3 to FT4 (FT3/FT4) were taken into account as continuous variables. Firstly, receiver-operating characteristic (ROC) curves based on TSH-CLNM, FT3-CLNM, FT4-CLNM, FT3/FT4-CLNM, TSH-LLNM, FT3-LLNM, FT4-LLNM, and FT3/FT4-LLNM were plotted. These continuous variables were converted into categorical variables by using an optimal cutoff value for analysis purposes. The subsequent step consists of categorizing the above variables according to the optimum cut-off values and using univariate analysis collectively with other risk variables to screen risk factors for CLNM or LLNM. Multivariate logical regression is used for constructing two model nomograms that separately forecast the contributing factors of CLNM or LLNM utilizing statistically significant variables from univariate analysis. Furthermore, to evaluate the predictive ability of the nomogram, we constructed a ROC curve and calculated the area under the curve (AUC). Then, a calibration plot was implemented to display the discrepancy between the results predicted by the nomogram and the actual outcome, which could demonstrate the accuracy of the predictive results. Ultimately, decision curve analyses (DCA) and clinical impact curves were performed for a more thorough assessment of the predictive model. All statistical analyses were conducted using R software 4.2.3 and p-value < 0.05 was considered statistically significant.

## Results

### Clinicopathological characteristics

Concerning the 6649 PTC patients for whom we eventually obtained adequate clinicopathological and ultrasound data. We randomly separated the 6068 PTC patients into the training group (n = 4247) and the internal validation group (n = 1821) in a 7:3 ratio to develop a predictive model along with enhanced model validation. Besides, the external test group included 582 patients from Yichun People’s Hospital. In the training cohort, patients underwent CLNM at a rate of 29.3%, compared to 29.8% and 37.1% of patients with CLNM in the internal validation and external test cohorts, respectively. Likewise, in the training cohort, patients suffered LLNM at a rate of 12.0%, while in the internal validation cohort and external test cohort, patients went through LLNM at rates of 11.8% and 15.5%, respectively. Chi-square was used to verify the consistency of all categorical variables and the Wilcoxon test was used to verify the consistency of all continuous variables between the training validation and the internal validation. Among all variables, age(p=0.055), gender (p=0.623), tumor size(p=0.129), mETE(minimal extrathyroidal extension), Multifocality, CLNM, LLNM, TSH, FT3, FT4,FT3/FT4 had no significantly statistical difference(P>0.05). There was no significant statistical difference among the two cohorts in general. More specific characteristics of enlisted patients are presented in **Table 1**.

**Table 1:** Clinical and pathological characteristics of patients.

characteristics	Training validation (4247)N(%)	Internal validation (1821)N(%)	P value	External test(582)N(%)
Age			0.055	
≥45	2272(53.5)	1023(56.2)		295(50.7)
<45	1975(46.5)	798(43.8)		287(49.3)
Gender			0.623	
Female	3198(75.3)	1382(75.9)		450(77.3)
Male	1049(24.7)	439(24.1)		132(22.7)
Tumor size (cm)			0.129	
≤1	2789(65.7)	1139(62.5)		288(49.5)
>1 and ≤2	1011(23.8)	471(25.9)		160(27.5)
>2 and ≤4	393(9.3)	188(10.3)		97(16.7)
>4	54(1.3)	23(1.3)		37(6.4)
mETE			0.550	
Negative	3071(72.3)	1337(73.4)		390(67.0)
Positive	1176(27.7)	484(26.6)		237(33.0)
Multifocality			0.990	
Negative	2888(68.0)	1238(68.0)		459(78.9)
Positive	1359(32.0)	583(32.0)		123(21.0)
Tumor location			0.034	
Isthmus	38(0.9)	28(1.5)		12(2.1)
Isthmus and left lobe	146(3.4)	77(4.2)		18(3.1)
Isthmus and right lobe	191(4.5)	92(5.1)		19(3.3)
Left lobe	1360(32.0)	540(29.7)		228(39.2)
Left and right lobe	815(19.2)	318(17.5)		66(11.3)
Right lobe	1603(37.7)	725(39.8)		223(38.3)
Isthmus left lobe and right lobe	94(2.3)	41(2.2)		16(2.7)
CLNM			0.707	
Negative	3001(70.7)	1278(70.2)		366(62.9)
Positive	1246(29.3)	543(29.8)		216(37.1)
LLNM			0.797	
Negative	3738(88.0)	1607(88.2)		492(84.5)
Positive	509(12.0)	214(11.8)		90(15.5)
TSH			0.143	
Median (quartile 1, quartile 3)	1.612(1.109,2.361)	1.659(1.128,2.458)		1.603(0.961,2.589)
FT3			0.104	
Median (quartile 1, quartile 3)	3.21(3.00,3.46)	3.20(2.97,3.45)		3.28(2.92,3.72)
FT4			0.058	
Median (quartile 1, quartile 3)	1.26(1.14,1.39)	1.24(1.12,1.36)		1.26(1.09,1.52)
FT3/FT4			0.445	
Median (quartile 1, quartile 3)	2.560(2.317,2.836)	2.593(2.353,2.843)		2.521(2.058,2.908)

**Figure 2:**
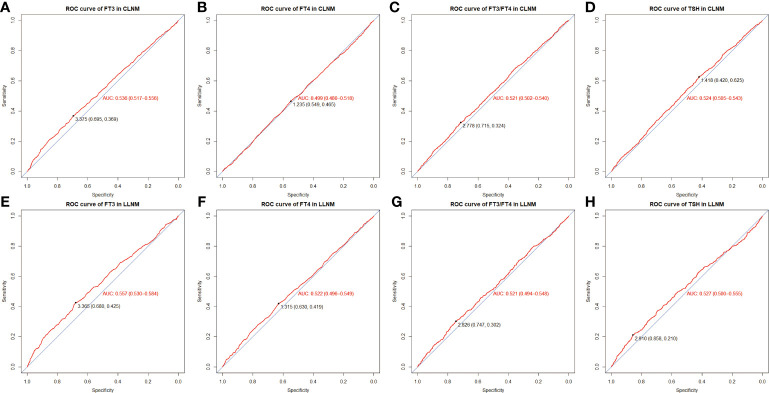
ROC curve and optimal cut-off value of FT3-CLNM **(A)**, FT4-CLNM **(B)**, FT3/FT4-CLNM **(C)**, TSH-CLNM **(D)** as well as FT3-LLNM **(E)**, FT4-LLNM **(F)**, FT3/FT4-LLNM **(G)** and TSH-LLNM **(H)**. CLNM, central lymph node metastasis; LLNM, lateral lymph node metastasis. FT3, free triiodothyronine; FT4, free thyroxine; TSH, thyroid stimulating hormone.

In order to conduct logistic regression analysis later, the continuous variables (including serum FT3, FT3/FT4, FT4, and TSH) were subsequently handled as categorical variables, with ROC curves based on TSH-CLNM, FT3-CLNM, FT4-CLNM, FT3/FT4-CLNM, TSH-LLNM, FT3-LLNM, FT4-LLNM, and FT3/FT4-LLNM drawn to figure out the best cutoff values (**Figure 2**). The optimal cut-off values were 1.418 (specificity 0.420%, sensitivity 0.625%), 0.375 (specificity 0.695%, sensitivity 0.369%), 1.235 (specificity 0,549%, sensitivity 0.465%), and 2.778 (specificity 0.715%, sensitivity 0.324%), respectively, for serum TSH-CLNM, FT3-CLNM, FT4-CLNM, and FT3/FT4-CLNM. Based on the mentioned above results, the variable serum TSH-CLNM was classified as ≥1.418 and <1.418, and serum FT3-CLNM, FT4-CLNM, and FT3/FT4-CLNM were graded as ≥0.375 and <0.375, ≥1.235 and <1.235, ≥2.778 and <2.778(mU/L). Also, the variable serum levels of TSH-LLNM, FT3-LLNM, FT4-LLNM, and FT3/FT4-LLNM were categorized as follows: ≥2.910 and <2.910, ≥3.365 and <3.365, ≥1.315 and <1.315, ≥2.826 and <2.826(mU/L) (**Figure 2**). Based on the American Joint Committee on Cancer (AJCC) staging manual and a thorough literature review, age was separated into ≥ 45 and < 45(years), Maximum tumor diameter was divided into 1, 1≥and <2, ≥2 and <4 and ≥4(cm) and the tumor location stratified into thyroid isthmus, isthmus and left lobe, isthmus and right lobe, left lobe, right lobe, left and right lobes, and total thyroid.

### Univariate and multivariate analysis of risk factors for CLNM

In an attempt to further screen suspect variables, we added all possible variables related to CLNM into univariate analysis after converting continuous variables into categorical variables. We found that Age, gender, tumor extension, tumor size, tumor location, serum FT3, multifocality (p <0.001), serum TSH (p =0.005), and FT3/FT4 (p =0.047) are all discovered to be risk factors for CLNM. (**Table 2**) Next, we put variables with p < 0.05 in univariate analysis into the multifactor logistic regression model. The multivariate analysis demonstrated that age <45 years (OR= 2.355, 95% CI: 2.035-2.726), male gender (OR= 1.741, 95% CI: 1.474-2.055, Maximum tumor diameter larger than 1 cm (1 cm≤tumor size < 2 cm, OR= 2.093, 95% CI: 1.780-2.461; 2 cm≤tumor size < 4cm, OR = 2.549, 95% CI: 2.026-3.206; tumor size≥ 4 cm, OR= 3.337, 95% CI: 1.871-5.952), tumor location (isthmus and right lobe, OR= 3.095, 95% CI: 1.007-9.512; left and right lobe, OR= 5.801, 95% CI: 1.972-17.062; right lobe, OR= 2.983, 95% CI: 1.113-7.992; total thyroid, OR= 3.674, 95% CI: 1.150-11.738), mETE (OR= 1.624, 95% CI: 1.390-1.896), and serum TSH ≥1.418 (OR= 1.242, 95% CI: 1.074-1.437) are crucial risk factors connected with CLNM while multifocality (OR= 0.914, 95% CI: 0.594-1.408), serum FT3 (OR= 0.975, 95% CI: 0.828-1.148), and FT3/FT4 (OR=1.132, 95% CI: 0.967-1.324) lost their predictive value. Based on the previously mentioned outcomes, we finally developed a multivariate logistic regression model with 6 dependent variables for predicting CLNM.

**Table 2:** Univariate and multivariate analyses of risk factors associated with CLNM in PTC patients.

Characteristics	CLNM(%) Absence(n=3001)	CLNM(%) Presence(n=1246)	P value	Multivariate analysis Adjusted OR(95%)	P value
Age			**<0.001**		
≥45	1435(47.8)	837(67.2)		1	
<45	1566(52.2)	409(32.8)		2.355(2.035-2.726)	**<0.001**
Gender			**<0.001**		
Female	2360(78.6)	838(67.3)		1	
Male	641(21.4)	449(36.0)		1.741(1.474-2.055)	**<0.001**
Tumor size(cm)			**<0.001**		
≤1	2156(71.8)	633(50.8)		1	
>1 and ≤2	607(20.2)	404(32.4)		2.093(1.780-2.461)	**<0.001**
>2 and ≤4	212(7.1)	181(14.5)		2.549(2.026-3.206)	**<0.001**
>4	26(0.9)	28(2.2)		3.337(1.871-5.952)	**<0.001**
mETE			**<0.001**		
Negative	2284(76.1)	787(63,2)		1	
Positive	717(23.9)	459(36.8)		1.624(1.390-1.896)	**<0.001**
Multifocality			**<0.001**		
Negative	2141(71.3)	747(60.0)		1	
Positive	860(28.7)	499(40.0)		0.914(0.594-1.408)	0.684
Tumor location			**<0.001**		
isthmus	33(1.1)	5(0.4)		1	
Isthmus and left	110(3.7)	36(2.9)		2.695(0.862-8.431)	0.088
Isthmus and right	140(4.7)	51(4.1)		3.095(1.007-9.512)	**0.049**
left	1037(34.6)	323(25.9)		2.372(0.884-6.367)	0.086
Left and right	467(15.6)	348(27.9)		5.801(1.972-17.062)	**0.001**
right	1152(38.4)	451(36.2)		2.983(1.113-7.992)	**0.030**
Isthmus left and right	62(2.1)	32(2.6)		3.674(1.150-11.738)	**0.028**
TSH			**0.005**		
<1.418	1261(42.0)	465(37.3)		1	
≥1.418	1740(58.0)	781(62.7)		1.242(1.074-1.437)	**0.003**
FT3			**<0.001**		
<3.375	2091(69.7)	797(64.0)		1	
≥3.375	910(30.3)	449(36.0)		0.975(0.828-1.148)	0.760
FT4			0.540	–	–
<1.235	1354(45.1)	575(46.1)		–	
≥1.235	1647(54.9)	671(53.9)		–	
FT3/FT4			**0.047**		
<2.778	2146(71.5)	853(68.5)		1	
≥2.778	855(28.5)	393(31.5)		1.132(0.967-1.324)	0.122

The p-value < 0.05, which is statistically significant. Significant results are given in bold.

### Univariate and multivariate analysis of risk factors for LLNM

Likewise, in order to screen out which variables are associated with the occurrence of LLNM, we added all possible variables related to LLNM into an univariate analysis model (**Table 3**). In this univariate logistic regression model, age, gender, tumor extension, tumor size, tumor location, multifocality, serum TSH, serum FT3, and CLNM (p < 0.001) are found to be statistically significant. Then, we put all variables with P < 0.05 into a multivariate logistic regression model to get the final multifactor regression equation. The results showed that age <45 years (OR= 1.440, 95% CI: 1.153-1.800, male gender (OR= 1.383, 95% CI: 1.090-1.755), maximal tumor diameter larger than 1 cm (1 cm ≤tumor size < 2 cm, OR= 1.966, 95% CI: 1.548-2.498; 2 cm ≤tumor size < 4 cm, OR = 4.387, 95% CI: 3.280-5.866;≥ 4 cm, OR= 5.490, 95% CI: 2.814-10.712), extension(OR= 1.765, 95% CI:1.421-2.193), multifocality (OR= 3.206, 95% CI:1.965-5.406), serum TSH ≥2.910 (OR= 1.242, 95% CI:1.074-1.437), and CLNM (OR= 4.512, 95% CI:3.627-5.613) increased the risk of LLNM. Based on the previously mentioned outcomes, we finally developed a multivariate logistic regression model with 7 dependent variables to predict LLNM.

**Table 3:** Univariate and multivariate analyses of risk factors for LLNM.

Characteristics	LLNM(%) Absence(n=3738)	LLNM(%) Presence(n=509)	P value	Multivariate analysis Adjusted OR(95%)	P value
Age			**<0.001**		
≥45	1801(48.2)	174(34.2)		1	
<45	1937(51.8)	335(65.8)		1440(1.153-1.800)	**0.001**
Gender			**<0.001**		
Female	2871(76.8)	327(64.2)		1	
Male	867(23.2)	182(35.8)		1.383(1.090-1.755)	**0.008**
Tumor size(cm)			**<0.001**		
≤1	2595(69.4)	194(38.1)		1	
>1 and ≤2	839(22.4)	172(33.8)		1.966(1.548-2.498)	**<0.001**
>2 and ≤4	270(7.2)	123(24.2)		4.387(3.280-5.866)	**<0.001**
>4	34(0.9)	20(3.9)		5.490(2.814-10.712)	**<0.001**
mETE			**<0.001**		
Negative	2799(74.9)	272(53.4)		1	
Positive	939(25.1)	237(46.6)		1.765(1.421-2.193)	**<0.001**
Multifocality			**<0.001**		
Negative	2653(71.0)	235(46.2)		1	
Positive	1085(29.0)	274(53.8)		3.260(1.965-5.406)	**<0.001**
Tumor location			**<0.001**		
Isthmus	36(1.0)	2(0.4)		1	
Isthmus and left lobe	136(3.6)	10(2.0)		0.369(0.065-2.090)	0.260
Isthmus and right lobe	172(4.6)	19(3.7)		0.539(0.101-2.880)	0.469
Left lobe	1248(33.4)	112(22.0)		1.550(0.337-7.126)	0.574
Left and right lobe	603(16.1)	212(41.7)		1.512(0.304-7.516)	0.613
Right lobe	1456(39.0)	147(28.9)		1.607(0.351-7.369)	0.541
Isthmus left lobe and right lobe	87(2.3)	7(1.4)		0.259(0.043-1.559)	0.140
CLNM			**<0.001**		
Negative	2839(75.9)	162(31.8)		1	
Positive	899(24.1)	347(68.2)		4.512(3.627-5.613)	**<0.001**
TSH			**<0.001**		
<2.910	3208(85.8)	397(78)		1	
≥2.910	530(14.2)	112(22)		1.242(1.074-1.437)	**0.003**
FT3			**<0.001**		
<3.365	2547(68.1)	300(58.9)		1	
≥3.365	1191(31.9)	209(41.1)		1.248(0.992-1.569)	0.059
FT4			0.288		
<1.315	1709(45.7)	220(43.2)		–	
≥1.315	2029(54.3)	289(56.8)		–	
FT3/FT4			0.164		
<2.826	2795(74.8)	366(71.9)		–	
≥2.826	943(25.2)	143(28.1)		–	

The p-value < 0.05, which is statistically significant. Significant results are given in bold.

### Development of nomogram model for LNM

**Figure 3:**
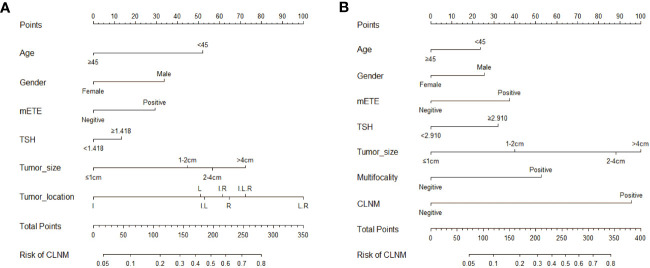
Nomograms based on clinical characteristics present the risk factors for CLNM **(A)** and LLNM **(B)**. mETE, minimal extrathyroidal extension; I, isthmus; L, left lobe; R, right lobe; I.L, Isthmus and left lobe; I.R, Isthmus and right lobe; I.L.R, Isthmus left lobe and right lobe; L.R, left and right lobe.

To further analyze the proportion of each independent risk factor for CLNM or LLNM, each independent risk factor was visualized in the form of a line, and the corresponding nomogram was created. **Figure 3** displays two new nomograms, where each variable matched a point on a scale of 0 to 100 according to the coefficient of regression of either CLNM or LLNM. Drawing a straight line based on the appropriate score for each variable, summing the total scores, and putting the results in the corresponding position on the total score serve as representations of the values of the variables. The nomogram revealed that maximal tumor size >4 cm was the most major factor to LLNM, while tumor location in both the left and right lobes was the most significant contributor to CLNM.

### Calibration and validation of the nomograms

**Figure 4:**
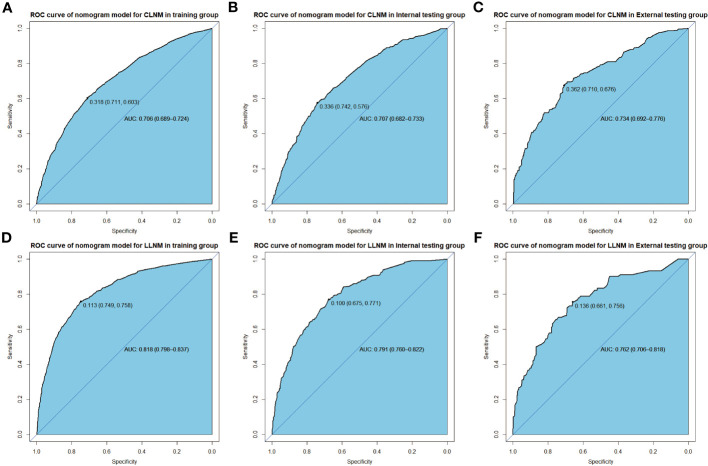
ROC curve shows nomogram predication model for CLNM in training cohort **(A)**, in internal validation cohort **(B)**, and in external test cohort **(C)**, in addition to predication model for LLNM in training cohort **(D)**, in internal validation cohort **(E)** and in external text cohort **(F)**.

ROC analysis is conducted on the training and verification cohort, and AUCs, also equivalent to C-statistic, are acquired to evaluate the model’s efficiency. The AUC value of the training cohort is 0.706 (sensitivity: 0.711, specificity: 0.630, cutoff value: 0.318), while the AUC values of the internal and external verification groups are 0.702 (sensitivity: 0.742, specificity: 0.576, cut-off value: 0.336) and 0.734 (sensitivity: 0.710, specificity: 0.676, cut-off value: 0.362), respectively (**Figures 4A–C**). It demonstrates in full the reliability of the CLNM prediction model in attempting to predictions. Similarly, in the forecasting model of LLNM, the AUC value for the training cohort is 0.818 (sensitivity: 0.749, specificity: 0.758, cut-off value: 0.113), and the AUC values for the internal validation and external test cohorts separately are 0.791 sensitivity: 0.675, specificity: 0.771, cut-off value: 0.100) and 0.762 (sensitivity: 0.661, specificity: 0.756, cut-off value: 0.136), respectively(**Figures 4D–F** **)**. The AUC values are unambiguous evidence of LLNM’s nomogram’s prediction capabilities.

**Figure 5:**
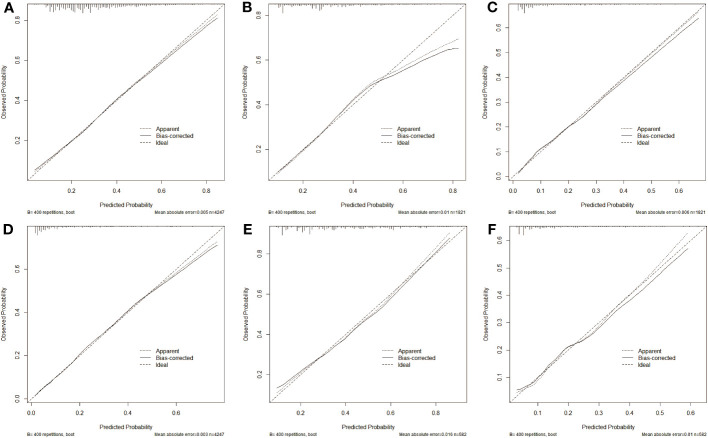
Calibration curve of predication model for CLNM in training cohort **(A)**, in internal validation cohort **(B)** and in external test cohort **(C)**, in addition to nomogram predication model for LLNM in training cohort **(D)**, in internal validation cohort **(E)** and in external test cohort **(F)**. The closer the three lines are, the higher accuracy of the model.

Additionally, calibration plots of our nomogram were carried out to verify the accuracy and repeatability of the nomogram model. Positive agreements were found between the actual and predicted probabilities of CLNM in the training cohort (mean absolute error MAE = 0.005), in the internal testing cohort (MAE= 0.01), and in the external testing cohort (MAE = 0.006, **Figures 5A–C**). For LLNM, the training cohort (MAE = 0.003), internal testing cohort (MAE = 0.016), and external testing cohort (MAE = 0.01, **Figures 5D–F**). The results generally achieve encouraging agreements between observation and prediction, with minimal variation shown in the calibration plots.

### Decision curve and clinical impact curve for clinical decision

**Figure 6:**
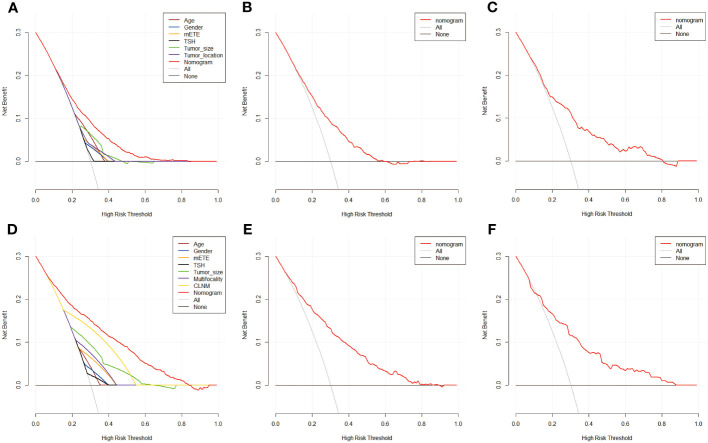
The decision curve of nomogram model and single factors of CLNM in training cohort **(A)**,in internal validation cohort **(B)** and in external test cohort **(C)**, in addition to nomogram predication model for LLNM in training cohort **(D)**, in internal validation cohort **(E)** and in external test cohort **(F)**. Decision curves of CLNM/LLNM risk factors present in the training cohort respectively. The gray line represents CLNM or LLNM positive and the horizontal black line represents CLNM or LLNM negative. When the red line is above the gray and black lines, the nomogram model possesses net return at this very risk threshold.

**Figure 7:**
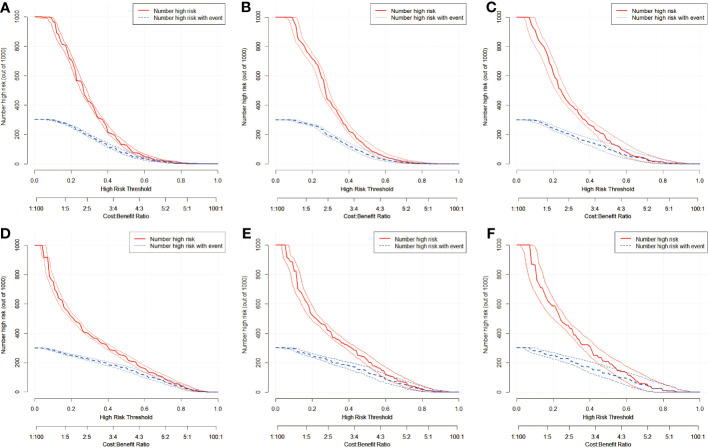
Clinical impact curves of predication model for CLNM in training cohort **(A)**, in internal validation cohort **(B)**, and in external test cohort **(C)**, in addition to nomogram predication model for LLNM in training cohort **(D)**, in internal validation cohort **(E)**, and in external test cohort **(F)**. The graph depicts the number of patients(out of 1000) identified as CLNM or LLNM positive at varying threshold probabilities, with the red curve representing the assumed positive cases, the blue curve indicating the true positive cases and the boundary lines curve represents 95%CI. The actual number of LNM positive and the number of LNM positive considered by the model are different when the diagnostic boundary is demarcated at different risk thresholds. The closer the two lines are, the higher net benefit of the predication model.

To further analyze the clinical application of the nomogram model, we established two clinical models to verify its efficiency. **Figures 6**, **7** show how the decision curves and clinical impact curves indicate the superiority of predictive models in clinical decisions with risk threshold, a dynamic variable that changes according to the clinicopathological characteristics of each patient. As seen in **Figure 6A**, when the risk threshold is between 0.1 and 0.7, the nomogram of CLNM has superior predictive power than single-factor models. Likewise, the LLNM prediction model performs better than the single-factor models when the risk threshold is between 0.1 and 0.8 (**Figure 6D**). The net return of the prediction model of CLNM or LLNM is larger than that of a none-treat or all-treat approach when the risk threshold is between 0.1 and 0.8 in the internal and external testing cohort, confirming that our models are quite effective. Clinical impact curve (CIC) analysis showed the clinical efficacy of the predictive model (**Figure 7**). When the risk threshold probability is greater than 45% of the total prediction score probability value in the training cohort for CLNM and 50% of the total prediction score probability value in the training cohort for LLNM, the CIC model determines that the high-risk population of LNM is highly matched with the actual population of LNM, which confirms the high clinical effectiveness of the nomogram prediction model.

## Discussion

In recent years, the increasing detection rate of thyroid cancer has attracted people’s attention, accompanied by the rapid development of imaging techniques. LNM is the most common form of PTC metastasis. However, the existing clinical examination methods are not sensitive to the diagnosis of occult lymph node metastasis (17, 19). The operation based on the preoperative evaluation of lymph node metastasis, instead of prophylactic central lymph node dissection may lead to tumor local recurrence and secondary surgery (22, 23). Although the prognosis of reoperation is still good, surgical complications such as laryngeal nerve injury and parathyroid gland injury are more likely to occur due to the hyperplasia and adhesion of scar tissue (24). Routine prophylactic central lymph node dissection(PCLND) proposed in Chinese thyroid diagnosis and treatment guidelines reduces the probability of cancer recurrence (14), but it also increases the risk of surgical complications (15). Whether to choose prophylactic lymph node dissection is currently a controversial area. Therefore, it is necessary to further explore more effective methods to predict the incidence of CLNM or LLNM in PTC patients.

There have been many studies using ultrasound features to predict LNM, and most diagnostic models have an AUC value of more than 0.7 (12, 25, 26). However, most studies only enlisted a few hundred to a few thousand samples, which cannot be regarded as high-quality evidence of evidence-based medicine research to change the existing clinical guidelines at present but provide a certain reference for future research. In our study, we recruited 6068 patients from two hospitals in southern China over a 10-year period with postoperative pathology confirmed PTC. Six variables, including age, sex, minimal extrathyroidal extension (mETE), serum TSH, tumor size, and location, were determined to be associated with CLNM by multivariate analysis. The risk factors for LLNM were subsequently selected as age, sex, mETE, multifocality, and serum TSH, as well as CLNM and tumor size. The AUC values of the CLNM-based nomogram reached 0.706, 0.707, and 0.734 in the three groups, and the AUCs of the LLNM-based nomogram reached 0.818, 0.791, and 0.762. Compared with single ultrasound imaging diagnosis (sensitivity, 0.33 for CLNM, 0.70 for LLNM) (27), our model improves the sensitivity of CLNM and LLNM diagnosis. Besides, the clinicopathological features of each patient are different, hence we developed clinical models, decision curves, and clinical impact curves to show the net benefit of our prediction model for LNM diagnosis at different risk thresholds. On the precondition of respecting patients’ wishes, our study provides a certain reference for individualized prophylactic central lymph node dissection.

Through a thorough literature search, we discovered that there is much literature exploring other efficient methods to forecast the risk of LNM for PTC patients (26, 28, 29). The nomograms were established and verified in all of the mentioned investigations, which exclusively focused on risk factors for CLNM. Most studies only enrolled a few hundred samples, but machine learning needs samples as training to increase accuracy and reduce errors. Therefore, the practical value of some studies is questionable. In addition to ultrasound, Zhou et al. used CT and clinicopathological features to establish a nomogram for Cervical LNM (30). The latest guidelines also mention that enhanced CT and MRI before surgery are recommended as auxiliary diagnoses for moderate and high-risk PTC suspected to have lymph node metastasis clinically (14). There may be more studies focusing on radiomics in the future. Our study established a nomogram based on clinicopathological traits and demographic data, not only with the aid of internal and external test cohorts but also screened risk factors for CLNM or LLNM. Besides, similar to ours, a multi-ethnic and multi-center retrospective study conducted by Feng et al., which was not limited to risk factors of CLNM in patients with PTC, had good concordance indices in the training cohort and internal and external cohorts, of which the C-indices were 0.733, 0.731, and 0.716 (31), respectively. However, there are some differences between our model and the model in Feng et al., and the possible reasons are as follows: (1) The data used by Feng et al. were primarily obtained from the SEER database, which does not include variables that can affect the occurrence of LNM, whereas the information we contained were obtained from our hospital and related hospitals; (2) The study included multiple races, whereas we focused on a single race; (3) Feng et al. viewed 55 years old as the age risk stratification point, whereas the current study divided the age into 45 years old; (4) Feng et al. established a nomogram based on cervical LNM risk assessment, while our study established two independent nomograms for CLNM and LLNM based on the corresponding variables.

There is some previous literature focusing on the impact of age on LNM (32, 33). In the retrospective study conducted by Zhang et al., multiple logistic regression analysis showed that the variable age was a risk factor for CLNM (34). Besides, studies have suggested that CLNM and LLNM are independent risk factors for the prognosis of PTC patients under the age of 45 (5). Consistent with the study by Li et al, our study presented that age <45, male, and tumor size are among the risk variables that may lead to CLNM in PTC patients. Interestingly, the influence of serum TSH on CLNM or LLNM cannot be neglected, according to our multivariate regression analysis. Some earlier investigations have demonstrated an association between high TSH levels and lymph node metastases, recurrence, and metastasis in PTC and it’s undisputed that the growth of thyroid tumors is influenced by serum TSH (35–37). However, there is insufficient evidence to conclude that serum TSH is a risk factor for LNM. We found that serum TSH levels above 1.418 (mU/L) also elevated the risk of CLNM and 2.910(mU/L) for LLNM, and their weight in the LLNM-based nomogram exceeded age and gender, which is consistent with the guidelines of the diagnosis and treatment of thyroid cancer that require exogenous thyroxine inhibition after surgery to maintain a low-level serum TSH, while some research did not identify a direct association between serum TSH and cervical LNM (13). Therefore, more research is needed to explore the relationship between serum TSH and cervical LNM. In addition, for the first time in our study, we noticed that tumor location (left and right thyroid lobes) is an independent risk factor for CLNM, while tumor location (thyroid isthmus) is a protective factor for CLNM. Interestingly, the correlation between tumor location and CLNM was inconsistent with previous studies (38, 39), which may be attributed to the distinction between multicentric and unicentric lesions. Most studies have proposed total thyroidectomy as an operative method for isthmic PTC. However, it is unclear whether isthmic PTC requires central lymph node dissection. American Thyroid Association (ATA) and European Thyroid Association (ETA) guidelines also do not specify the operative method for isthmic PTC (15, 40). More high-quality research is still needed in the future

Despite providing some original views and good findings, our study still has some limitations. Firstly, our study is a retrospective study. Retrospective studies generally contain more biases and mistakes than prospective studies; secondly, given the model’s constrained scope, the predictive model may only apply to PTC patients but not to other subtypes of thyroid cancer. Lastly, due to the limited number of patient samples in our institution, there is inevitably a certain error in statistical analysis. In order to obtain more objective results, a larger sample size is needed as support in the future

## Conclusion

In summary, we found age, preoperative serum TSH, and tumor size as common risk indicators for CLNM and LLNM, with tumor location being the most weighted variable for CLNM and tumor size for LLNM. We established two corresponding nomograms to visually display the independent risk variables related to lymph node metastasis in PTC patients and drew clinical decision curves and impact curves to help clinicians rationalize the management and treatment of PTC patients.

## Data availability statement

The raw data supporting the conclusions of this article will be made available by the authors, without undue reservation.

## Ethics statement

The studies involving humans were approved by Ethics Committee of the Second Affiliated Hospital of Nanchang University (No.Review(2011)No.(041)). The studies were conducted in accordance with the local legislation and institutional requirements. Written informed consent for participation was not required from the participants or the participants’ legal guardians/next of kin in accordance with the national legislation and institutional requirements.

## Author contributions

YL designed this study. HJ, WL, HS, ZS, and TY performed the data collection and validation. WL and HJ analyzed the data and completed the figures. DZ, WL, and FX were in charge of the writing. JP and YL provided the study material and made critical revisions to the manuscript. All authors contributed to the article and approved the submitted version.
